# A review of the diversity in taxonomy, definitions, scope, and roles in forensic medicine: implications for evidence-based practice

**DOI:** 10.1007/s12024-018-0031-6

**Published:** 2018-10-02

**Authors:** Putri Dianita Ika Meilia, Michael D. Freeman, Herkutanto, Maurice P. Zeegers

**Affiliations:** 1 Care and Public Health Research Institute (CAPHRI), Maastricht University Medical Center+, Universiteitssingel 40, 6229 ER Maastricht, the Netherlands; 2 Department of Forensic Medicine and Medicolegal Studies, Faculty of Medicine, University of Indonesia, Jl. Salemba Raya No. 4, Salemba, Jakarta Pusat, 10430 Indonesia

**Keywords:** Forensic medicine, Forensic pathology, Clinical forensic medicine, Medico-legal studies, Evidence-based practice

## Abstract

The scope, roles, and tasks of forensic medicine and forensic medical experts currently vary widely between countries and legal systems, which has resulted in barriers to organization, standard setting, and quality assurance for practice in forensic medicine, including for reporting. The legal fact finder is thus confronted with variability in the quality, structure, and content of forensic medical reports. We sought to define and categorize the scope, methods, and practices that fall under the description of forensic medicine, the various issues encountered in current forensic medical practice, and the potential role of evidence-based practice in forensic medicine. We searched electronic databases and reviewed relevant articles, as well as conducting personal correspondences with forensic medical practitioners around the world, to obtain a description of current forensic medical practice. The terms forensic medicine, legal medicine, medical jurisprudence, medico-legal services, forensic pathology, and clinical forensic medicine are used with mixed interpretations in different countries. The systems and services rendered are not uniform either. The methods used by forensic medical practitioners are not always evidence-based, or based on standardized methods, and vary greatly between experts and centers. There are also no universally accepted guidelines to prepare a standard and admissible report. The lack of a uniform system in forensic medicine creates difficulties in assessing the development and performance of forensic medicine as a distinct discipline. To prepare evidence-based forensic medical reports, generally accepted guidelines are necessary.

## Introduction

*Forensic medicine* is a nonspecific term used to describe a broad area of medical practice that is concerned with the intersection between medicine and the law. It comprises of the expert application of medical knowledge, technology, and analysis to assist legal proceedings*. Forensic* as a word is thought to have its origin from the Latin word *forensis*, meaning “of or before the forum” (a forum being a public place where, among other things, disputes were settled in ancient Rome) [1].

Depending on where it is practiced, forensic medicine is also known variously as legal medicine, medical jurisprudence, and medico-legal practice. The uncertainty as to what forensic medicine should be called reflects the lack of uniformity regarding what it is that comprises forensic medicine practice. Aside from the common element of using medical knowledge, methods, and technology for legal purposes, the scope, roles, and tasks of forensic medical experts vary widely between countries and legal systems. In the United States, for example, forensic medicine is synonymous with the practice of forensic pathology [2–7], which largely consists of autopsies conducted in cases of suspicious or unobserved deaths. In European countries and the United Kingdom, however, forensic medicine has a far broader scope and includes investigation of sexual assault, medical negligence, police practices, and other matters in which medicine and the law cross paths [8].

The variety of definitions and scope of work that may be considered to fall under the general category of forensic medicine has resulted in barriers to the organization, standard setting, and quality assurance for practice in forensic medicine [9]. Such tasks are further complicated by the retrospective nature of forensic medical work, which often results in probabilistic conclusions that cannot be measured against a gold standard, in contrast with clinical medicine which results in ascertainable outcomes that are followed prospectively. The role of evidence-based practice, which is an essential component of clinical medicine, is also comparatively less well defined in forensic medicine, which is largely comprised of experience-based practice [10].

In the present review, we have sought to define and categorize the scope, methods, and practices that fall under the description of forensic medicine in various countries around the world. Furthermore, the various issues encountered in current forensic medical practice and the potential role of evidence-based practice in forensic medicine were also identified.

## Methods

A literature survey was conducted by searching electronic databases of PubMed, EMBASE, ClinicalKey, MEDLINE, Wiley Online, BMJ, as well as Google Search and Google Scholar for articles up to December 2017. The search terms used were as follows: “forensic medicine”, “forensic medical services”, “forensic pathology”, “clinical forensic medicine”, “legal medicine”, “medico-legal” (various spelling variations), “autopsy”, and “forensic report”. We restricted the language to English, but no restriction on format was applied. The search results were sorted by relevance. We evaluated all relevant articles and included them in this review.

In addition, we also conducted personal correspondence (by email or face-to-face) with forensic medical practitioners from various countries and regions via professional and personal networks. We asked questions regarding forensic medical practice in their country and the current practice in forensic medicine.

## Results

### Taxonomy

The term *forensic medicine* is employed in the Netherlands, Belgium, Germany, France, Sweden, Norway, Egypt, Saudi Arabia, Turkey, Iran, Bangladesh, Japan, China, Indonesia, and Australia [11–21]. The name is not used universally, however, and in other countries the term *legal medicine* is used instead of *forensic medicine* [22–25]. Other terms with similar or overlapping meaning include *medical jurisprudence* and *medico-legal services* [20, 26, 27]*.* In some countries, including the USA and Canada *forensic medicine* and *forensic pathology* (i.e. medico-legal autopsy practice) are interchangeable, and there is no single formal designation for other applications of medical knowledge in a legal setting. In the UK and some other countries (including the USA, Canada, and India) the term *clinical forensic medicine* is used to describe forensic services rendered by clinicians from various non-forensic disciplines (surgeons, emergency physicians, gynecologists, etc.) [7, 28–31]. As a further taxonomic complication, opinions vary on whether *forensic medicine* and *legal medicine* are synonymous or whether they are two separate entities, with *forensic medicine* relating to criminal law and *legal medicine* concerned with civil and tort law [32].

Due to the inconsistency of the terms used to describe similar practices, it is unsurprising that the scope and role of *forensic medicine* vary between countries. For the sake of consistency, in the following sections, the term *forensic medicine* will be used to refer to the discipline, and *forensic medical practitioner* will be used to designate the individual practicing within the discipline.

### Scope and role of forensic medicine

The systems and services rendered by forensic medicine are not uniform and vary between countries. Generally, the different systems can be classified into two main categories of forensic medical service. The first can be labeled as the “integrated services” type [18, 19, 33, 34]. In this type of service, the forensic medical practitioner conducts investigations of death and injury associated with suspected criminal acts. The service includes the conduct of medico-legal autopsy (i.e. forensic pathology practice), and the examination of living victims of physical and sexual assault, which are activities that fall under the umbrella term of clinical forensic medicine. The integrated services type of forensic medical practice may also include consultations pertaining to medical ethics and negligence, and the conduct of forensic laboratory examinations, such as those relevant to forensic serology or forensic genetics. To qualify as a forensic medical practitioner in an integrated system, medical doctors are required to undergo additional postgraduate or specialist education [21, 25, 35], varying by country. The basic principles of forensic medicine may also be taught at the undergraduate level, particularly in countries that require all medical doctors to perform forensic medical examinations, if necessary, due to the shortage of specially trained forensic medical practitioners [21, 25, 33, 36–38].

The second category of forensic medical services can be described as the “divided type” of service. In the divided service the variety of tasks provided by a single practitioner in the integrated system are handled by different practitioners within forensic medicine. Forensic pathologists, typically working within a medical examiner/coroner system (i.e. the USA, the UK, Australia, and New Zealand) exclusively handle death investigation, typically associated with the performance of an autopsy or external examination of a decedent [3, 5, 39, 40]. Clinical forensic medical examinations, and sometimes medico-legal consultations as well, are conducted by general practitioners, police surgeons (a general practitioner with a particular assignment and contract with the police), or other relevant medical specialists (for example, a specialist in obstetrics and gynecology in cases of sexual abuse or emergency physicians for trauma victims). In the divided services system, clinical forensic medicine is not linked with forensic pathology and has variable recognition as a subfield within forensic medicine [4, 6, 7, 26, 28–31, 41–43]. In countries with a divided services system principles and practice of forensic medicine are neither taught to medical undergraduates nor routinely provided as postgraduate education. The principles of forensic examination are either included in the curriculum of postgraduate training in relevant disciplines (emergency medicine, gynecology, nursing, etc.) or not at all [6, 29]. Table 1 describes the types of forensic service system by country.

**Table 1:** Type of forensic medical services in different countries

Integrated service	Divided service
Argentina [44] Bangladesh [13] Belgium [12, 45] China [37, 46] Denmark [2, 47, 48] Egypt [3, 18] Finland [2, 49] France [2, 15, 50, 51] Germany [14, 52, 53] Hungary [54, 55] Iceland [56] Indonesia [37] Iran [21] Italy [24] Kuwait [3] Poland [57] Portugal [2, 23, 25] Romania [58] Russia [59–61] Saudi Arabia [3, 19] Spain [2, 22] Sri Lanka [33, 37, 62] Sweden [8, 16] Switzerland [63] Tunisia [35] Turkey [20]	Australia and New Zealand [37, 64] Canada [65, 66] Hong Kong [67] India [31, 68, 69] Japan [36, 37, 70] The Netherlands [11, 26, 71] Norway [17] Singapore [37] South Africa [28, 72] UK [2, 3, 5, 26, 29, 73–77] USA [2–4, 6, 7, 41]

Forensic medicine, as it is variously practiced, is a hybrid discipline, relying on principles drawn from a variety of core and adjunct disciplines, including medicine, and specifically pathology, pharmacology, and toxicology. In cases of injury resulting from mechanical trauma, principles of applied physics, including injury biomechanics and ballistics, are also used. Another adjunct discipline that is increasingly relied on in forensic medicine is forensic epidemiology, which applies population-based data and methods as a form of evidence-based causation analysis in forensic medicine [78, 79]. In select cases, practices from disciplines more appropriately considered to be part of forensic sciences rather than forensic medicine are employed or relied on, including serology, genetics, dactylography (fingerprint analysis), forensic anthropology, and forensic odontology.

### Current situation and practice of forensic medicine

Despite a long history of practice, with evidence of investigations dating back to early civilizations [80], forensic medicine remains one of the least known and most misunderstood specialties of medicine. Forensic medicine, and forensic pathology in particular, is a comparatively rarely chosen profession [39, 81], with many medical undergraduates considering it gruesome, outside of the clinical setting, and with long and unpredictable working hours and insufficient job appreciation compared to other specializations (monetary and otherwise). There are currently no data on the number of practitioners working in the field of forensic medicine, which may be due to the differences in the definitions used for forensic medical services, titles of practitioners, and the educational and practice systems in the countries listed in Table 1.

The wide variety of types of cases handled by forensic medical practitioners include, among others, cases of alleged murder/homicide, suicide, physical assault/abuse, accidental injury causation, sexual assault/abuse, poisoning, medical negligence claims, the cause of food and blood-borne illness, competency and dangerousness, and disaster victim identification. The annual number of criminal and civil investigations and cases that involve or require forensic medical expertise worldwide, much less in various countries is not known. This lack of data is despite the existence of WHO’s World Health Statistics that includes data regarding a variety of forensic-related health topics (e.g. road traffic injuries, violence, and substance abuse). The potential number of cases globally that are likely to involve forensic medical investigation based on data available in early 2016 is shown in Table 2 [82].

**Table 2:** WHO world health statistics of forensic-related health topics (2016)

Topic	Global Incidence
Death due to substance abuse (esp. alcohol)	3,300,000
Death due to road-traffic injuries	1,250,000
Non-fatal injuries due to road traffic accidents	20,000,000 – 50,000,000
Homicide	475,000
Suicide	800,000

Along with the increase of public awareness regarding the role of forensic medicine in society, largely due to romanticized portrayals in popular media [83–85], the demand for high-quality service is increasing. Despite the vital role of forensic medical expert opinion in the justice system, however, the methods used by forensic medical practitioners are not always evidence-based or based on standardized methods. Many forensic medical practitioners currently rely more on experience and individual customary practices in formulating their expert opinion [10]. Furthermore, many operational principles and procedures used by forensic medical practitioners have not been standardized and therefore may vary greatly between experts and centers [86].

The difference in available resources also contributes to the variety of services rendered. Some centers in high-resource countries complement conventional autopsy with post-mortem imaging techniques [87, 88], while others have explored the usefulness of post-mortem biochemistry investigation in establishing the cause-of-death [89]. On the other hand, resource-poor countries often resort to cheaper or simpler alternatives of the conventional autopsy, such as verbal autopsy [90] or minimally-invasive autopsy [91, 92]. Furthermore, forensic medical practitioners must also consider various non-medical factors, especially in settings or populations with a certain degree of aversion towards autopsy due to social or religious reasons [3, 93, 94]*.* This variation leads to difficulties in comparing and reviewing the performance and output of forensic medical service between nations and even between centers in the same country*.*

There have been several efforts to initiate a harmonization of rules in forensic medicine, particularly forensic autopsies. One example is the 1999 European Harmonization of Medico-legal Autopsy Rules effort [95], which was updated in 2014 [96]. We were unable, however, to find publications about experiences in using, or the level of success of, the harmonized rules so far. Furthermore, although an accreditation/certification guideline for forensic pathology services exists [97], it has limited scope, both regarding countries which implement it as well as the type of forensic medical services subject to accreditation.

Forensic medical expert opinions are provided in either oral or written forms. Generally, a written report contains the opinion of the expert on the issues involved in the case evaluation, as the core element of the involvement of the forensic medical practitioner in the case [98]. Unlike clinical/hospital records, which are secondary to the medical procedure performed by the clinician (and are sometimes considered as “a necessary evil” [98]) written reports are the primary product of forensic medical practice. They are also the principal means of communication between the forensic medical practitioner and legal practitioners as their customers.

The first published work about the methods to be adopted in preparing medico-legal reports was written in sixteenth-century France by Ambroise Paré [80, 99], which was part of several volumes on the subject of forensic medicine. The development of forensic medicine in different parts of the world occurred at a different pace and through varied processes so that the reports produced by forensic medical experts also differ in scope and quality. A literature search using the keywords *forensic medical report, forensic medicine report, clinical forensic report,* and *autopsy report* revealed numerous samples of forensic reports, but no standardized guidelines regarding how to prepare a standard and admissible forensic medical report. Although an example of guidelines for an autopsy report exists [100]*,* the majority of publications are concerned with the writing of reports in forensic psychiatry/psychology, where the issue is the mental health state of the accused, criminal responsibility, community danger, and competence to stand trial [98, 101–103]. There are various samples of customary examination forms produced by different agencies and institutions available on the internet, including forms for sexual assault victim examination and autopsy report forms. A list of some examples, and the search keywords used to find them, is available in the Appendix.

There are countless variations in the format, content, and terms used in the reports [86], not only between countries or centers but between experts as well. One element that is commonly missing from the reports is an explanation of the methods used by the expert to come to a conclusion, the justification for the use of the methods, and supporting references to scientific papers and databases. There is often a conclusory leap from the objective findings directly to the conclusion, without any explanation about the basis of the opinion rendered, or whether the said opinion is based on best-available evidence at all.

## Discussion

The lack of a uniform taxonomy and system in forensic medicine creates difficulties in assessing the development and performance of forensic medicine as a distinct discipline. The fact that differences in practice are both intra- and inter-country makes the development of generally accepted codes of conduct and specific practice guidelines problematic. It is therefore unsurprising that current practices in forensic medicine tend to be experienced-based, passed from generation to generation of forensic medical practitioners at individual facilities. To overcome these difficulties, we propose that the terms *forensic medicine* and *forensic medical practitioner* be more widely and more uniformly used. The term *forensic medicine* should be used as an umbrella term for any and all medical practices that involve the intersection of medicine and law, whereas the term *forensic medical practitioner* defines practicing medical consultants who have undergone specially designed education and training in forensic medicine. Furthermore, the terms *forensic pathology, clinical forensic medicine, forensic epidemiology,* etc. should be used to denote specific services rendered as part of forensic medicine. The use of a uniform taxonomy could facilitate the development of universally accepted practice standards despite local specificities (e.g. legal systems, medical education systems, and available resources).

Despite the existence of various codes of conduct for expert witnesses [104–106], the lack of common definitions and standardized techniques in forensic medicine presents an obstacle in following those codes. This difficulty is particularly apparent when two or more expert witnesses have dissenting views, which they say are supported by different assessment methods that they have relied on in formulating their opinions. Without common practice guidelines, it is practically impossible for legal fact-finders to decide which opinion is closer to the truth.

Additionally, forensic medical services are now starting to lose their geographic/national boundaries, e.g. in cases involving expatriates visiting/living in other countries [107] and multi-center/multi-national research studies. The product of forensic medical practitioners of one country will have to withstand the scrutiny of their peers from other countries. Without a uniform understanding of what comprises “forensic medicine,” it is difficult to imagine smooth cooperation between forensic medical practitioners, in addition to being a source of frustration to users of forensic medical services.

Over the past several decades the forensic sciences have been promoted to the forefront of public consciousness, via media coverage, as well as television shows and movies, as a fail-proof discipline that is practiced by infallible experts, in which all complex mysteries are solved in an hour-long episode via sophisticated technology and highly accurate inference. This fictitious representation of forensic sciences in general, and forensic medicine specifically, has resulted in the so-called “CSI-effect” among judicial fact-finders [83–85], in which experts are expected to be able to provide valid and reliable expert opinions with a 100% degree of certainty. In reality, expert opinion is only as reliable as the inferential methods and historical data used to arrive at the opinion. Because of the critical role that expert opinions play in legal proceedings, it is equally critical that the opinions be formulated with transparent methods based on sound methodology. The use of “bad science”, with faulty methodology or inappropriate supporting data, can introduce random error as well as systematic bias in the process of expert opinion formulation. Causal analysis in forensic medicine can be a somewhat untidy process, requiring a sufficient understanding of medical matters, causal methodology, and legal standards for admissibility of expert opinion [108].

Evidence-based practice has yet to be explicitly implemented in forensic medicine, particularly when it comes to the semi-subjective nature of causal evaluation. There exists a certain degree of reluctance in forensic medicine to supplement experience-based practice with evidence-based practice because of the belief that it will complicate the formulation and interpretation of expert opinions. Enigmatically, it is the semi-subjective nature of experience-based practice that makes the addition of evidence-based practices critical to the validity of future practice to avoid bias due to the lack of the relevant experience, as well as the use of improper methodology and data.

Forensic medical expert opinions presented to legal fact-finders should be combined in a comprehensive expert opinion, with comprehensible language and logic that is easily understood by legal fact-finders. Expert opinions that are inappropriately formulated, with a confusing structure or language, or cannot be easily understood by legal practitioners and other consumers of forensic medical opinions, can be misleading and lead to the wrong legal decision [109]. Due to the lack of uniformity and standardization of forensic medical reports, quality assessment and comparison of the reports can be problematic for the judicial fact finder.

To prepare forensic medical reports that are evidence-based, generally accepted guidelines are necessary. These guidelines should be in the form of recommendations, meant to apply to a wide range of matters requiring forensic medical analysis, which are demonstrably accepted by forensic medical practitioners, both intra-nationally and internationally. The justification of the use of particular methods, data, or literature should be provided, to enable readers to follow the reasoning of the report author. This practice will allow the reader to understand how the author arrived at a certain conclusion, given the specific circumstances of the case. In such a manner the level of transparency in forensic medicine will be enhanced, and thus improve the average quality of forensic medical reports by facilitating peer review efforts [9, 110].

Currently, there exist no universally accepted or international guidelines for writing a forensic medical report. The lack of generally accepted standards and evidence-based practices has an adverse impact on the reliability of the outcome of forensic medical investigations [10, 109, 111]. The results of forensic medical investigations are provided to the justice system most commonly in the form of a written report, which is sometimes accompanied by oral testimony. Individual forensic medicine centers are responsible for setting their standards for reports, and thus there are many variations in the format, content, and terms seen in forensic medical reports [86]. Because each forensic medicine center has its own report format, and within each center each forensic medical practitioner has his/her own style of writing, forensic medical reports are not generally subjected to quality assurance processes such as peer review and comparison to gold standards. The legal fact finder is thus confronted with variability in the quality, structure, and content of forensic medical reports, the result of which is a threat to the reliability of legal decision-making. This problem could be overcome by developing universally accepted guidelines, which will hopefully provide common practice and report-writing standards despite a diversity of local specificities, thereby promoting evidence-based practice in forensic medicine.

## Conclusion

The lack of a uniform taxonomy and system in forensic medicine creates difficulties in assessing the development and performance of forensic medicine as a distinct discipline. Furthermore, the paucity of generally accepted standards and evidence-based practices has an adverse impact on the reliability of the outcome of forensic medical investigations, particularly regarding the semi-subjective nature of causal evaluation. Hence, it is necessary to develop universally accepted guidelines that provide common practice standards despite a diversity of local specificities to improve the reliability of forensic medical expert opinions.

## Key points

1. Taxonomy and the scope of forensic medicine vary between countries and legal systems.
2. The methods used are not always evidence-based or based on standardized methods.
3. It is difficult to assess the performance of forensic medicine as a distinct discipline.
4. Evidence-based practice should be implemented, especially in causal evaluation.
5. To prepare evidence-based reports, generally accepted guidelines are necessary.

## Appendix

**Table 3:** Websites containing examples of forensic medical examination forms

*Keyword: forensic medical report*	
http://www.caloes.ca.gov/GrantsManagementSite/Documents/2-925%20Forensic%20Medical%20Report,%20Nonacute%20Child-Adolescent%20Sexual%20Abuse%20Examination.pdf	
http://www.fris.org/Resources/ToolKit-Disabilities/PDFs/Section-B/B11.%20Sexual%20Assault%20Forensic%20Medical%20Examination.pdf	
http://www.sahealth.sa.gov.au/wps/wcm/connect/public+content/sa+health+internet/health+topics/health+conditions+prevention+and+treatment/rape+and+sexual+assault/forensic+medical+examination+after+a+sexual+assault	
http://www.icmr.nic.in/dhr/pdf/1%20DHR%20Forensic%20Medical%20Manual%20Sexual%20Assault.pdf	
https://aifs.gov.au/publications/role-forensic-medical-evidence-prosecution-adult-sexual-assault/export	
http://www.forensicindia.com/pmr.pdf	
*Keyword: forensic medicine report*	
https://emedicine.medscape.com/article/1718019-overview	
*Keyword: clinical forensic report*	
https://www.ncbi.nlm.nih.gov/pubmed/20182738	
https://www.abpp.org/files/page-specific/3356%20Forensic/22_--Forensic%20Report%20Checklist.pdf	
*Keyword: autopsy report*	
http://www.autopsyfiles.org/	
https://www.utmb.edu/meded/year4/autopsy_4th_year/autopsyreportsample.pdf	
